# Immunity and Protective Efficacy of Mannose Conjugated Chitosan-Based Influenza Nanovaccine in Maternal Antibody Positive Pigs

**DOI:** 10.3389/fimmu.2021.584299

**Published:** 2021-03-04

**Authors:** Sankar Renu, Ninoshkaly Feliciano-Ruiz, Veerupaxagouda Patil, Jennifer Schrock, Yi Han, Anikethana Ramesh, Santosh Dhakal, Juliette Hanson, Steven Krakowka, Gourapura J. Renukaradhya

**Affiliations:** ^1^Food Animal Health Research Program, Ohio Agricultural Research and Development Center, Wooster, OH, United States; ^2^Department of Veterinary Preventive Medicine, Wooster, OH, United States; ^3^The Department of Veterinary Biosciences, College of Veterinary Medicine, The Ohio State University, Columbus, OH, United States

**Keywords:** chitosan nanoparticle, mannose, swine influenza virus, intranasal vaccination, immune response, maternally derived antibodies, pigs

## Abstract

Parenteral administration of killed/inactivated swine influenza A virus (SwIAV) vaccine in weaned piglets provides variable levels of immunity due to the presence of preexisting virus specific maternal derived antibodies (MDA). To overcome the effect of MDA on SwIAV vaccine in piglets, we developed an intranasal deliverable killed SwIAV antigen (KAg) encapsulated chitosan nanoparticles called chitosan-based NPs encapsulating KAg (CS NPs-KAg) vaccine. Further, to target the candidate vaccine to dendritic cells and macrophages which express mannose receptor, we conjugated mannose to chitosan (mCS) and formulated KAg encapsulated mCS nanoparticles called mannosylated chitosan-based NPs encapsulating KAg (mCS NPs-KAg) vaccine. In MDA-positive piglets, prime-boost intranasal inoculation of mCS NPs-KAg vaccine elicited enhanced homologous (H1N2-OH10), heterologous (H1N1-OH7), and heterosubtypic (H3N2-OH4) influenza virus-specific secretory IgA (sIgA) antibody response in nasal passage compared to CS NPs-KAg vaccinates. In vaccinated upon challenged with a heterologous SwIAV H1N1, both mCS NPs-KAg and CS NPs-KAg vaccinates augmented H1N2-OH10, H1N1-OH7, and H3N2-OH4 virus-specific sIgA antibody responses in nasal swab, lung lysate, and bronchoalveolar lavage (BAL) fluid; and IgG antibody levels in lung lysate and BAL fluid samples. Whereas, the multivalent commercial inactivated SwIAV vaccine delivered intramuscularly increased serum IgG antibody response. In mCS NPs-KAg and CS NPs-KAg vaccinates increased H1N2-OH10 but not H1N1-OH7 and H3N2-OH4-specific serum hemagglutination inhibition titers were observed. Additionally, mCS NPs-KAg vaccine increased specific recall lymphocyte proliferation and cytokines IL-4, IL-10, and IFNγ gene expression compared to CS NPs-KAg and commercial SwIAV vaccinates in tracheobronchial lymph nodes. Consistent with the immune response both mCS NPs-KAg and CS NPs-KAg vaccinates cleared the challenge H1N1-OH7 virus load in upper and lower respiratory tract more efficiently when compared to commercial vaccine. The virus clearance was associated with reduced gross lung lesions. Overall, mCS NP-KAg vaccine intranasal immunization in MDA-positive pigs induced a robust cross-reactive immunity and offered protection against influenza virus.

## Introduction

Swine influenza is an acute respiratory disease of pigs caused by swine influenza A virus (SwIAV) (1). Pigs are naturally vulnerable to IAV-associated with secondary bacterial infections (2). Swine IAV is an economic threat to the global pig industry (3). Commonly circulating SwIAV strains in swine population are H1N1, H1N2, and H3N2 (4). In the United States, periodically human infections are occurred from some of the SwIAVs (5). In last two decades, triple reassortant SwIAVs have been isolated from pigs (5), and its association with human infections have also been documented (6, 7). The most recent is the 2009 pandemic H1N1 SwIAV spillover to humans (8, 9). Therefore, vaccination of pigs is a common practice to reduce the influenza burden in swine industry and to avoid the risk of zoonotic transmission to humans (10). The SwIAV vaccine inoculated into sows protects the herd from infection and heightens the transfer of maternally-derived antibodies (MDA) to offspring through colostrum (11, 12). However, a number of studies have revealed that MDA offered various levels of protection against IAV infection in piglets (2, 11, 12). In weaned piglets, MDA interferes with parenteral administered killed/inactivated influenza virus vaccines, resulting in poor induction of antibody responses and documented evidence of vaccine-associated enhanced respiratory disease (2, 13–15).

The MDA inhibits the vaccine-induced IgG antibody and does not interfere with the secretory IgA (sIgA) antibody production (16). Intranasally (IN) administered inactivated IAV vaccine in mice overcomes the MDA interference and provides complete protection in offspring (16). Influenza viruses use nasal mucosa as a main entry site. Effective vaccines delivered IN trigger the mucosal immunity and offer the frontline defense against the infection (17). Further, IN vaccination activates the B and T cells in the nasal-associated lymphoid tissues and induce specific antibody and cell-mediated immune responses. However, to achieve effective IN immunization, novel vaccine formulation(s) containing innovative vaccine delivery vehicle and/or adjuvant (18, 19) are needed.

Chitosan is a biocompatible polymer, and its protonated positively charged amino groups electrostatically interact with negative charged mucus sialic acid and epithelial surfaces to become mucoadhesive vehicle (20, 21). Hence, we used chitosan nanoparticles (CS NPs) as a mucosal vaccine delivery carrier for the poultry and swine vaccines to combat infectious diseases (22–25). In protein antigens encapsulated CS NPs, treated immune cells *in vitro* demonstrate upregulated multiple Toll-like receptors (TLRs), Th1 and Th2 cytokines gene expression (25). In SwIAV killed antigen loaded CS NPs treated dendritic cells (DCs) observed enhanced secretion of innate, pro-inflammatory and Th1 cytokines, and in IN vaccinated pigs, induction of enhanced cross-reactive mucosal immunity has been observed (26).

The calcium-dependent (C-type) lectin family mannose receptor (MR) is a carbohydrate binding protein, primarily expressed by the DCs and macrophages (27). The MR binds to mannosylated protein and the antigens uptaken through MR are efficiently processed and presented through major histocompatibility pathways by DCs (27, 28). Mannose ligand is internalized by DCs through receptor-mediated endocytosis (29). *In vitro*, mannan ligand-coated nanoparticles readily binds to MR expressing cells and internalized (30). A study revealed that mannose ligand in mannosylated CS NPs interact with MR on the surface of macrophages and facilitate its uptake (31). *In vivo*, glycosylated nanoparticles rapidly shuttle to the follicular DCs network and are concentrated in germinal centers of lymph nodes thereby triggering the innate immune-mediated recognition pathway and promotes antigen-specific responses (32). For these reasons, the MR receptor on cells is a possible target for vaccine delivery (27). To test this hypothesis, we conjugated mannose ligand with chitosan (mCS) and formulated killed SwIAV antigen (KAg) encapsulated mCS NPs (mCS NPs-KAg) vaccine. The efficacy of IN-administered mCS NPs-KAg and KAg encapsulated CS NPs (CS NPs-KAg) vaccine in MDA-positive pigs were determined and compared to an intramuscularly (IM) administered multivalent commercial SwIAV vaccine as a positive control.

## Materials and Methods

### Preparation of Influenza Viruses and Source of Commercial SwIAV Vaccine

The field isolates of IAVs—A/Swine/OH/FAH10-1/10 (H1N2-OH10), A/Swine/OH/206/20 (H1N1-OH7), and A/Turkey/OH/2053/20 (H3N2-OH4) were grown in Madin-Darby Canine Kidney epithelial (MDCK) cells (33). The virus-rich cell free supernatant was clarified using sucrose density gradient ultracentrifugation and the viral pellet was suspended in phosphate-buffered saline (PBS). Viruses were inactivated using binary ethyleneimine and the inactivation efficiency was confirmed by re-culture in MDCK cells (33) and as henceforth it is called KAg. The virus titers were analyzed in MDCK cells (33). The protein content in inactivated virus was tested using a micro-BCA protein assay kit (Thermo Scientific, MA, USA) as per the company recommendations. Commercial inactivated SwIAV vaccine (FluSure XP^®^) was obtained from Zoetis (MI, USA) and used as per the company recommendations. The FluSure XP^®^ is a multivalent vaccine containing H1N1, H1N2, and H3N2 SwIAVs.

### Formulation of Experimental Vaccines

Chitosan-based NPs encapsulating KAg (CS NPs-KAg) and mannosylated chitosan-based NPs encapsulating KAg (mCS NPs-KAg) vaccines were prepared using an ionic gelation method as previously described (24). For mannose-conjugated chitosan (mCS) preparation, 40 mg each of mannose (Sigma, MO) and sodium triacetoxyborohydride (Sigma, MO) mixture in 0.2 M borate buffer was slowly added into 200 mg chitosan [1% (w/v)] suspension (25) under magnetic stirring for 72 h at 56°C (34). The mCS was dialyzed against milli-Q-water for 48 h. Both chitosan and mannose modified chitosan were dissolved in 1% acetic acid solution. Twenty milligrams of mCS or chitosan were added into 20 ml milli-Q-water under magnetic stirring, pH was adjusted to 4.3 and mixed with 2 mg KAg (H1N2-OH10) in 3-(N-morpholino) propanesulfonic acid (MOPS) buffer pH 7.4. Followed by tripolyphosphate [1% (w/v) (Sigma, MO)] 5 mg in 10 ml milli-Q-water was added dropwise and the mCS NPs-KAg or CS NPs-KAg vaccines were obtained after centrifugation at 10,976 × g for 30 min, washed, dispersed in milli-Q-water and used for vaccination. Both the vaccines had ~80% antigen encapsulation efficiency and characterized as described previously (24, 25). Both the chitosan-based vaccine formulations were freshly prepared and used in animals.

### Experimental Plan

Three genetically related pregnant sows (Yorkshire x Landrace) were vaccinated with FluSure XP^®^ vaccine at 2 and 5 weeks before farrowing as per the manufacturer's instruction, and the naturally born piglets were weaned at 3 weeks of age and transported to the Ohio Agricultural Research and Development Center (OARDC) biosafety level-2 animal holding facility. From each sow litter received over 60% piglets which were well-grown and looked very healthy and randomized to have at least one piglet from each sow present in every experimental group. Blood samples from piglets were collected and tested for SwIAV-specific antibody titers. The MDA-positive piglets (*n* = 19) were randomly distributed into a five experimental groups as follows: (i) Mock (no vaccination and no challenge, *n* = 3); (ii) Mock-challenge (no vaccination and challenge, *n* = 4); (iii) FluSure XP^®^ vaccine (*n* = 4); (iv) CS NPs-KAg vaccine (10^7^ TCID_50_ equivalent of KAg from H1N2-OH10 virus to each piglet, *n* = 4); and (v) mCS NPs-KAg vaccine (10^7^ TCID_50_ equivalent of KAg from H1N2-OH10 virus to each piglet, *n* = 4).

Experimental piglets at age 3 weeks received the mCS NPs-KAg or CS NPs-KAg vaccine through both the nostrils using a spray mist delivery device (Prima Tech USA, NC). Commercial FluSure XP^®^ vaccine was administered IM as per the company recommendation. Pigs received a booster dose of vaccine like the prime dose 3 weeks later. Two weeks after booster vaccination, experimental vaccinates (except mock control group) were challenged (Ch) with a heterologous H1N1-OH7 virus 6 × 10^6^ TCID_50_ (50% IN and 50% intratracheal after anesthetizing the animals) (33). Pigs were monitored daily for clinical signs (35) and euthanized 6 days after challenge by anesthetizing followed by exsanguination. Blood and nasal swab samples were collected before and after prime and boost vaccinations. At necropsy, along with blood and nasal swab samples, bronchoalveolar lavage (BAL) fluid, lung samples for preparing lung lysate (represents lung parenchyma), tracheobronchial lymph nodes (TBLN) in DMEM for isolating mononuclear cells (MNCs) and pieces of TBLN tissues in RNA later were collected, processed and stored as described previously (33). Gross lung lesions were scored based on the presence of virus affected purple red consolidation in each lung lobe. The final lung lesion score of each pig was obtained by averaging all the scores recorded in dorsal and ventral lobes. Images of dorsal and ventral views of the lungs were captured from all the pigs.

### Antibody Titration

The pre-titrated KAg extracted from H1N2-OH10, H1N1-OH7, or H3N2-OH4 viruses were coated in duplicate (35) in 96-well plates (Greiner bio-one, Monroe, NC, USA) and incubated overnight at 4°C. Plates were washed with PBS Tween-20 (0.05%) (PBST) and blocked with 5% skim milk powder in PBST for 2 h, at room temperature (RT). After plates washed, serially diluted nasal swab, lung lysate and BAL fluid samples were analyzed for sIgA; and serum, lung lysate and BAL fluid samples for IgG antibodies by adding to marked duplicate wells and incubated overnight at 4°C. Plates were washed and horseradish peroxidase conjugated goat anti-pig IgA (Bethyl Laboratories, Montgomery, TX) or goat anti-pig IgG (KPL, Gaithersburg, MD) antibodies were added and incubated for 2 h, at RT. Plates were washed and 1:1 mixture of peroxidase substrate solution B and TMB peroxidase substrate (KPL, Gaithersburg, MD) was added, and after 10–20 min the reaction was terminated with 1 M phosphoric acid solution. The optical density (OD) values were measured at 450 nm in ELISA Spectramax microplate reader (Molecular devices, CA), and samples corrected OD values were attained after subtraction of the blank value.

Hemagglutination inhibition antibody titers in serum samples collected at day 6 post challenge against H1N2-OH10, H1N1-OH7, and H3N2-OH4 viruses were analyzed as reported earlier (36). Briefly, 10-fold serially diluted heat inactivated sera in triplicates was added to eight HA units of virus and incubated at 37°C for 1 h. The hemagglutination inhibition titers were calculated by using the 50% endpoint method.

### Cell Proliferation Analysis

The isolated TBLN MNCs at day post challenge (DPC)-6 was subjected to cell proliferation analyses as reported previously (33, 35). Briefly, 1 × 10^6^ cells/well in triplicate in 10% FBS containing RPMI medium was plated in a 96 well flat-bottom plate (Greinerbio-one, NC). Cells were either unstimulated or stimulated with 0.1 multiplicity of infection (MOI) vaccine (H1N2-OH10) and challenge (H1N1-OH7) viruses for 72 h at 37°C in 5% CO_2_ incubator. The 20 μl MTS + PMS solution (Promega, WI) was added to each well before 4 h of 72 h incubation, and the OD at 490 nm was recorded using the ELISA Spectramax microplate reader. Stimulation index was calculated by dividing OD of stimulated from OD of unstimulated cells of the same animal.

### Quantitative Reverse Transcription PCR (qRT-PCR) Analysis

Total RNA was extracted from TBLN stored in RNAlater using TRIzol reagent (Invitrogen, Carlsbad, CA). The cDNA syntheses (25) was attained from 2 μg of total RNA (37), and the target cytokine IL-4, IL-10, and IFNγ, and internal control β-actin (Supplementary Table 1) (24, 38) genes expression were achieved using the SYBR Green Supermix kit (Bio-Rad Laboratories, CA) by qRT-PCR (Applied Biosystems, CA). Gene expression in fold changes was calculated as described (37).

### Challenge Virus Titration

The procedure for virus titration was followed as described previously (33). Briefly, nasal swab collected at DPC-4 and DPC-6, BAL fluid and lung lysate samples collected at DPC-6 were 10-fold serially diluted in TPCK-trypsin containing serum-free DMEM medium, added into monolayer of MDCK cells and incubated for 36 h at 37°C in 5% CO_2_ incubator. Cells were fixed and immunostained with IAV nucleoprotein specific primary antibody (CalBioreagents, CA) followed by AlexaFluor 488 conjugated goat anti-mouse IgG (H+L) secondary antibody (Life Technologies, CA). Immunofluorescence signal was observed in a fluorescent microscope (IX51, Olympus, Tokyo, Japan) and the virus titers were calculated.

### Statistical Analyses

Two-way ANOVA followed by a Bonferroni test was used for statistical analyses of ELISA data using the GraphPad Prism 8 (GraphPad Software, Inc., CA). The remaining experimental data were examined by one-way ANOVA followed by Tukey's *post-hoc* comparison test. Date were presented as mean of three to four pigs ± standard error mean (SEM) of each experimental group. Results were considered statistically significant when *p* < 0.05.

## Results

### Prime-Boost Immunization of MDA-Positive Pigs With mCS NPs-KAg Vaccine Prior to Challenge Increased the Cross-Reactive sIgA Antibody Response

**Figure 1:**
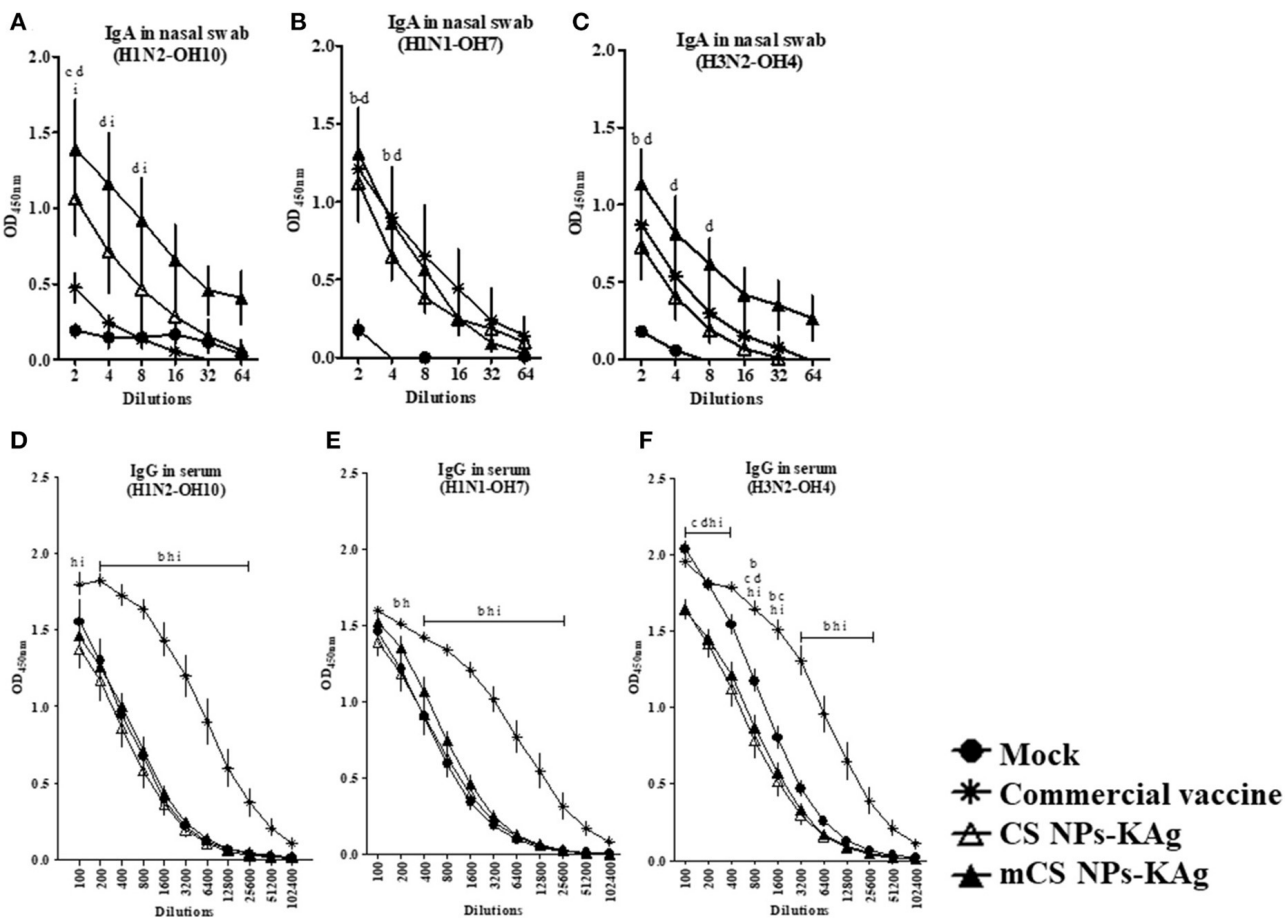
Mannose conjugated CS NPs based influenza (mCS NPs-KAg) vaccine augmented cross-reactive secretory IgA while the commercial flu vaccine boosts specific IgG antibodies in MDA-positive pigs. Pigs were vaccinated twice with mCS NPs-KAg or CS NPs-KAg vaccines (containing H1N2-OH10 virus) intranasally or commercial vaccine (containing H1N1, H1N2 and H3N2 viruses) intramuscularly. On the day post vaccination 35 nasal swab and blood samples collected were subjected to antibody analysis. Secretory IgA antibody response in nasal swab against **(A)** H1N2-OH10; **(B)** H1N1-OH7; and **(C)** H3N2-OH4 viruses, and IgG antibody response in serum against **(D)** H1N2-OH10; **(E)** H1N1-OH7; and **(F)** H3N2-OH4 viruses were analyzed by ELISA. Data represent the mean value of three to four pigs ± SEM at all indicated dilutions. Statistical analysis was carried out using two-way ANOVA followed by a Bonferroni test. Each letter on the line graph indicates the significant difference between the groups at the marked sample dilution: b, c and d indicate the difference between mock group compared to commercial vaccine, CS NPs-KAg, and mCS NPs-KAg, respectively; h and i indicate the difference between commercial vaccine compared to CS NPs-KAg and mCS NPs-KAg, respectively. A *p* < 0.05 was considered statistically significant.

All the weaned piglets born to vaccinated mothers used in this experimental trial had high levels of SwIAV specific MDA in serum, with no significant difference between the groups (Supplementary Figure 1). In mCS NPs-KAg vaccine inoculated MDA-positive pigs after two doses of vaccination at day post vaccination 35 detected enhanced homologous (H1N2-OH10) and heterosubtypic (H3N2-OH4) IAV-specific sIgA antibody levels in nasal swabs at all the tested dilutions compared to mock, commercial and CS NPs-KAg vaccinates (Figures 1A,C). Compared to commercial SwIAV vaccine, both mCS NPs-KAg and CS NPs-KAg vaccinates increased H1N2-OH10 virus-specific sIgA antibody levels in nasal swabs, while mCS NPs-KAg vaccine increased level was significantly (*p* < 0.05) higher (Figure 1A). mCS NPs-KAg and commercial vaccinates had significantly (*p* < 0.05) increased H1N1-OH7 and H3N2-OH4 viruses-specific sIgA antibody levels in nasal swab compared to mock pigs (Figures 1B,C). On the other hand, commercial SwIAV vaccinates significantly (*p* < 0.05) increased H1N2-OH10, H1N1-OH7, and H3N2-OH4 viruses-specific IgG antibody levels in serum than all the other groups were observed (Figures 1D–F).

### mCS NPs-KAg and CS NPs-KAg Vaccinates Augmented Cross-Reactive sIgA Antibody Response Following Challenge Infection

**Figure 2:**
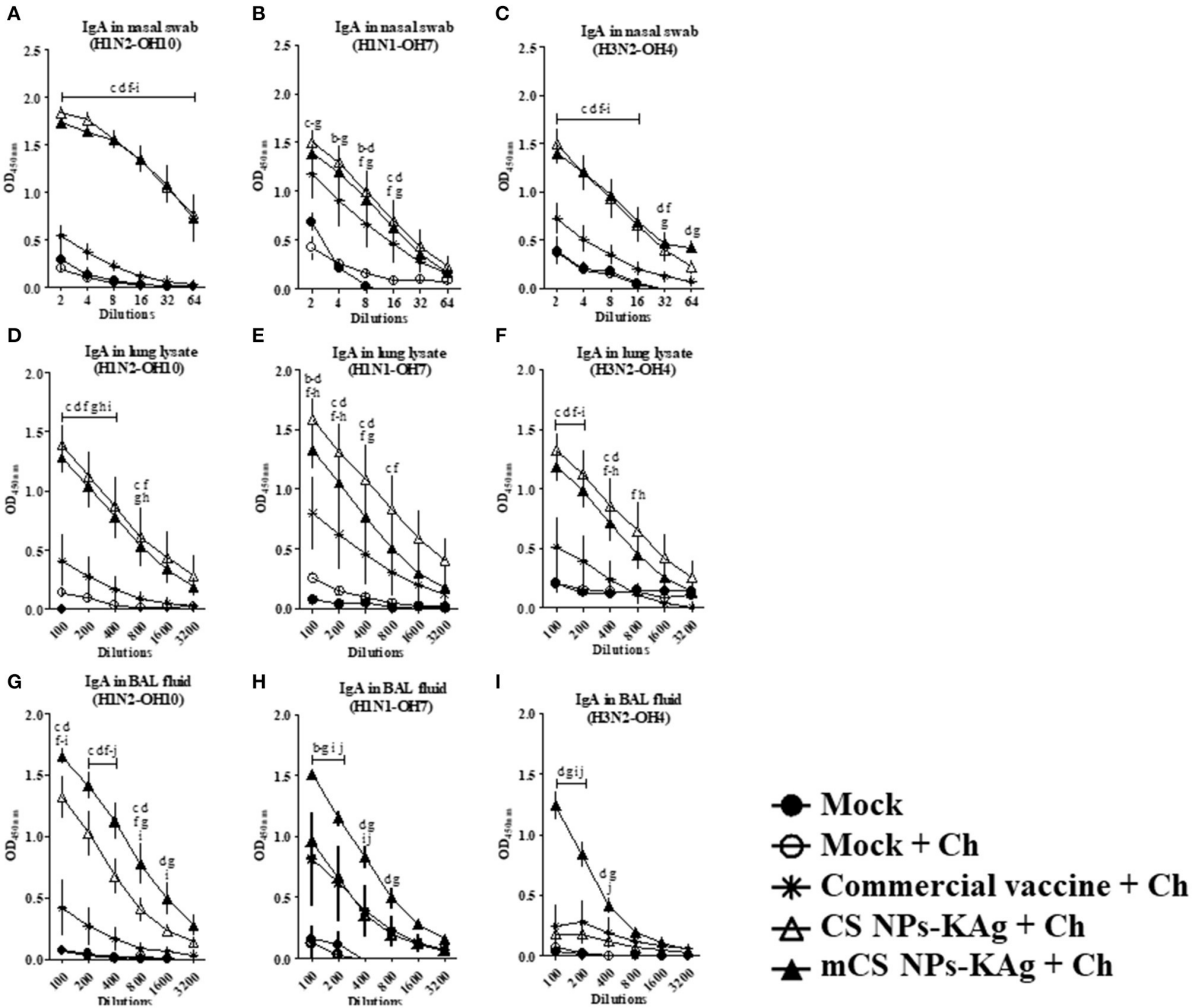
Mannose-conjugated and unconjugated CS NPs based influenza (mCS NPs-KAg and CS NPs-KAg) vaccinates elicited higher cross-reactive secretory IgA antibody response in MDA-positive pigs post challenge infection. Pigs were prime-boost vaccinated with mCS NPs-KAg or CS NPs-KAg vaccine (containing H1N2-OH10 virus) intranasally or commercial vaccine (containing H1N1, H1N2, and H3N2 viruses) intramuscularly and at day post vaccination 35 challenged with a heterologous H1N1-OH7 virus. On day six post-challenge secretory IgA antibodies in nasal swab, lung lysate, and BAL fluid samples against **(A,D,G)** H1N2-OH10; **(B,E,H)** H1N1-OH7; and **(C,F,I)** H3N2-OH4 viruses were analyzed by ELISA. Data represent the mean value of three to four pigs ± SEM at all indicated dilutions. Statistical analysis was carried out using two-way ANOVA followed by a Bonferroni test. Each letter on the line graph indicates the significant difference between the groups at the marked sample dilution: b, c and d indicate the difference between mock group compared to commercial vaccine + Ch, CS NPs-KAg + Ch, and mCS NPs-KAg + Ch, respectively; e, f, and g indicate the difference between mock + Ch group compared to commercial vaccine + Ch, CS NPs-KAg + Ch and mCS NPs-KAg + Ch, respectively; h and i indicate the difference between commercial vaccine + Ch compared to CS NPs-KAg + Ch and mCS NPs-KAg + Ch, respectively. j indicate the difference between CS NPs-KAg + Ch compared to mCS NPs-KAg + Ch. A *p* < 0.05 was considered statistically significant. Ch, Challenge.

In SwIAV challenged pigs, mCS NPs-KAg and CS NPs-KAg vaccinates had a significantly (*p* < 0.05) higher H1N2-OH10 and H3N2-OH4 virus-specific sIgA antibody levels in nasal swab at tested all the dilutions (2 to 64) compared to other groups including commercial influenza vaccine (Figures 2A,C). Although, in mCS NPs-KAg and CS NPs-KAg vaccinates increased H1N1-OH7 virus specific sIgA antibody levels in nasal secretions was detected compared to other experimental groups, statistical significance (*p* < 0.05) was reached compared to the mock and mock-challenge groups (Figure 2B). As in the nasal swab samples, the H1N2-OH10 and H3N2-OH4 viruses-specific sIgA antibody levels in lung lysate was also significantly (*p* < 0.05) augmented by mCS NPs-KAg and CS NPs-KAg vaccines compared to all the other groups (Figures 2D,F). In addition, unlike the sIgA level in the nasal cavity, the CS NPs-KAg vaccinates had significantly (*p* < 0.05) increased H1N1-OH7 virus specific sIgA antibody levels in lung lysates compared to commercial vaccine (Figure 2E). The H1N2-OH10 virus specific sIgA antibody level in BAL fluid was significantly (*p* < 0.05) increased in both mCS NPs-KAg and CS NPs-KAg vaccinates over other groups including commercial vaccine received animals (Figure 2G). It is important to note that compared to all the groups including CS NPs-KAg vaccinates, the mCS NPs-KAg vaccinates had a remarkably (*p* < 0.05) increased H1N2-OH10, H1N1-OH7, and H3N2-OH4 virus-specific sIgA antibody levels in BAL fluid (Figures 2G–I).

### mCS NPs-KAg and CS NPs-KAg Vaccinates had Increased Cross-Reactive IgG Antibody Response After Challenge Infection in Serum, Lung Lysate, and BAL Fluid

**Figure 3:**
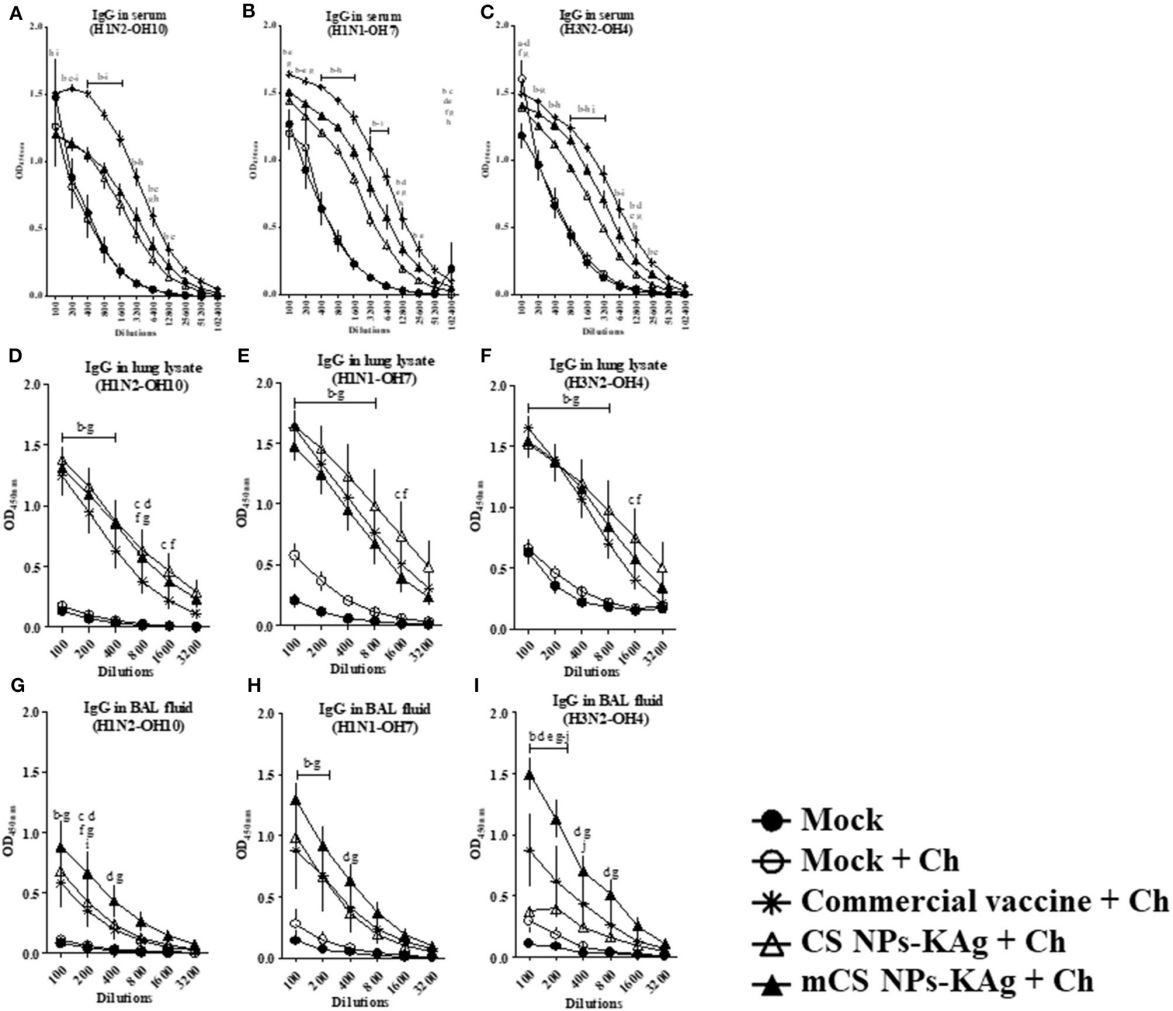
Commercial swine flu vaccine increased the cross-reactive serum IgG, and mannose-conjugated and unconjugated CS NPs influenza (mCS NPs-KAg and CS NPs-KAg) vaccines augmented IgG antibody response in lung lysate and BAL fluid of MDA-positive pigs. Pigs were vaccinated with mCS NPs-KAg or CS NPs-KAg vaccine (containing H1N2-OH10 virus) intranasally or commercial vaccine (containing H1N1, H1N2, and H3N2 viruses) intramuscularly and at day post vaccination 35 challenged with a H1N1-OH7 virus. On day 6 post challenge IgG antibody response in serum, lung lysate and BAL fluid samples against **(A,D,G)** H1N2-OH10; **(B,E,H)** H1N1-OH7; and **(C,F,I)** H3N2-OH4 viruses were analyzed by ELISA. Data represent the mean value of three to four pigs ± SEM at all indicated dilutions. Statistical analysis was carried out using two-way ANOVA followed by a Bonferroni test. Each letter on the line graph indicates the significant difference between the groups at the marked sample dilution: b, c and d indicate the difference between mock group compared to commercial vaccine + Ch, CS NPs-KAg + Ch, and mCS NPs-KAg + Ch, respectively; e, f, and g indicate the difference between mock + Ch group compared to commercial vaccine + Ch, CS NPs-KAg + Ch, and mCS NPs-KAg + Ch, respectively; h and i indicate the difference between commercial vaccine + Ch compared to CS NPs-KAg + Ch and mCS NPs-KAg + Ch, respectively; j indicate the difference between CS NPs-KAg + Ch compared to mCS NPs-KAg + Ch. A *p* < 0.05 was considered statistically significant. Ch, Challenge.

In the serum of mCS NPs-KAg, CS NPs-KAg and commercial SwIAV vaccinates, significantly (*p* < 0.05) increased IgG antibody levels specific to H1N2-OH10, H1N1-OH7, and H3N2-OH4 viruses compared to mock and mock-challenge groups was observed. The commercial flu vaccine received animals had significantly (*p* < 0.05) increased IgG antibody levels compared to CS NPs-KAg and mCS NPs-KAg vaccinates (Figures 3A–C). In CS NPs-KAg, mCS NPs-KAg, and commercial vaccinates a significant (*p* < 0.05) increase in H1N2-OH10, H1N1-OH7, and H3N2-OH4 viruses specific IgG antibody levels in lung lysate sample compared to mock and mock-challenge groups was evident (Figures 3D–F). Interestingly, compared to pigs given commercial vaccine in CS NPs-KAg vaccinates detected numerically increased H1N2-OH10, H1N1-OH7, and H3N2-OH4 viruses specific IgG antibody levels in lung lysates, even though this increase was not statistically significant (Figures 3D–F). Conversely, in the BAL fluid, like the observed enhanced sIgA antibody, the IgG antibody levels in mCS NPs-KAg vaccinates was also significantly (*p* < 0.05) increased against H3N2-OH4, H1N2-OH10, and H1N1-OH7 viruses at some of the tested dilutions compared to commercial vaccine (Figures 3G–I). Furthermore, in mCS NPs-KAg vaccinates, significantly (*p* < 0.05) increased H3N2-OH4 virus specific IgG antibody level in BAL fluid was detected when these values were compared to values obtained from the BAL fluids of CS NPs-KAg vaccinates (Figure 3I).

### Hemagglutination Inhibition (HI) Titers in the Serum of mCS NPs-KAg and CS NPs-KAg Vaccinates Were Increased Against the Vaccine Virus but Not Against Variant IAVs

**Figure 4:**
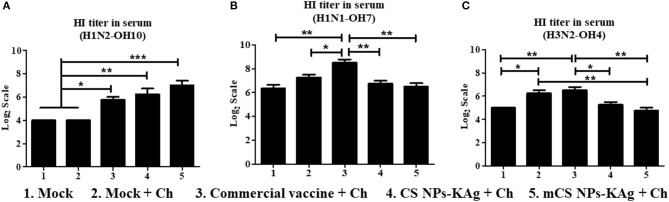
Hemagglutination inhibition (HI) antibody titers in MDA-positive pigs vaccinated with mannose-conjugated and unconjugated CS NPs based influenza (mCS NPs-KAg and CS NPs-KAg) or commercial flu vaccine and challenged at day post vaccination 35 with the H1N1-OH7 virus. On day six post challenge HI antibody titer in serum against **(A)** H1N2-OH10; **(B)** H1N1-OH7; and **(C)** H3N2-OH4 viruses were measured. Data represent the mean value of three to four pigs ± SEM. Statistical analysis was carried out using one-way ANOVA followed by Tukey's *post hoc* comparison test. Asterisk refers to statistical difference between the two indicated groups (*p < 0.05, **p < 0.01, and ***p < 0.001). Ch, Challenge.

The mCS NPs-KAg (*p* < 0.001), CS NPs-KAg (*p* < 0.01) and commercial vaccines (*p* < 0.05) significantly increased the H1N2-OH10 virus specific HI titers in serum compared to values obtained in mock and mock-challenge pigs (Figure 4A). Compared to commercial vaccine, both mCS NPs-KAg and CS NPs-KAg vaccinates had increased serum HI titers against H1N2-OH10 virus by 2.3 and 1.4 times, respectively, but these data were not statistically significant (Figure 4A). Commercial vaccine received animals had significantly higher H1N1-OH7 (*p* < 0.01) and H3N2-OH4 (*p* < 0.05) viruses specific HI titers compared to mCS NPs-KAg and CS NPs-KAg vaccinates (Figures 4B,C). In addition, commercial vaccine induced significantly higher H1N1-OH7 virus specific HI titers than those recorded in mock (*p* < 0.01) and mock-challenge (*p* < 0.05) pig groups (Figure 4B). Both the commercial vaccine (*p* < 0.01) and mock-challenge (*p* < 0.05) pigs had a significantly increase HI titers against H3N2-OH4 virus compared to mock group. In addition, mock-challenge group had increased (*p* < 0.01) tires compared to mCS NPs-KAg group (Figure 4C).

### mCS NPs-KAg and CS NPs-KAg Vaccines Increased the Cell-Mediated Immunity in The Local Cytokine Gene Expression in TBLN

**Figure 5:**
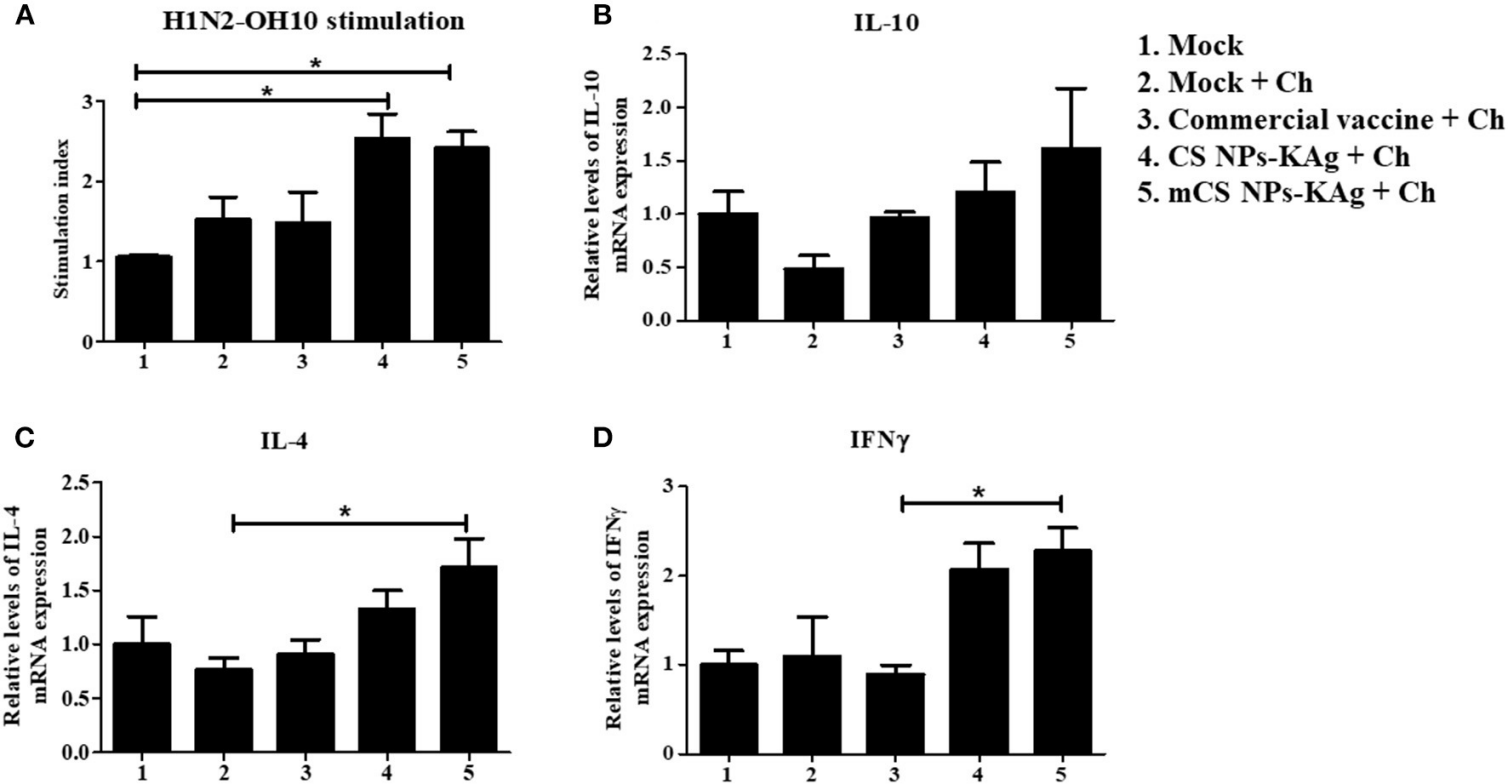
Augmented cell-mediated immune response in MDA-positive pigs vaccinated with mannose-conjugated and unconjugated CS NPs based influenza (mCS NPs-KAg and CS NPs-KAg) vaccines and not commercial flu vaccine. At day 6 post challenge infection with H1N1-OH7 virus the isolated MNCs of tracheobronchial lymph nodes (TBLN) were stimulated with vaccine virus (H1N2-OH10). **(A)** Lymphocytes proliferation stimulation index in TBLN was analyzed by ELISA. TBLN tissues stored in RNAlater were analyzed by qRT-PCR for the expression of mRNA of **(B)** IL-10; **(C)** IL-4; and **(D)** IFNγ. Data represent the mean value of three to four pigs ± SEM. Statistical analysis was carried out using one-way ANOVA followed by Tukey's *post hoc* comparison test. Asterisk refers to statistical difference between the two indicated groups (*p < 0.05). Ch, Challenge.

The mCS NPs-KAg and CS NPs-KAg vaccines induced a virus-specific proliferative cellular immune response in TBLN MNCs at DPC-6 as determined by analyzing the lymphocyte proliferation index (Figure 5A). The vaccine (H1N2-OH10) virus stimulated lymphocyte stimulation index in TBLN of both the mCS NPs-KAg and CS NPs-KAg vaccinates was significantly (*p* < 0.05) higher compared to mock group (Figure 5A). However, the vaccinated experimental groups did not have significant increase in challenge (H1N1-OH7) virus specific lymphocyte stimulation index values (data not shown). We analyzed the different cytokine gene expression profiles as correlates with the mCS NPs-KAg and CS NPs-KAg vaccines enhanced humoral and cell mediated immune responses. Both the mCS NPs-KAg and CS NPs-KAg vaccinates had increased expression of Th2 (IL-4), Th1 (IFNγ), and IL-10 cytokine mRNA levels compared to all the other experimental groups including commercial vaccine (Figures 5B–D). While only in mCS NPs-KAg vaccinates the upregulated IL-4 and IFNγ cytokine gene expressions were significantly (*p* < 0.05) higher compared to mock-challenge and commercial vaccine groups, respectively (Figures 5C,D).

### mCS NPs-KAg and CS NPs-KAg Vaccines Reduced/Cleared the Challenge SwIAV

**Figure 6:**
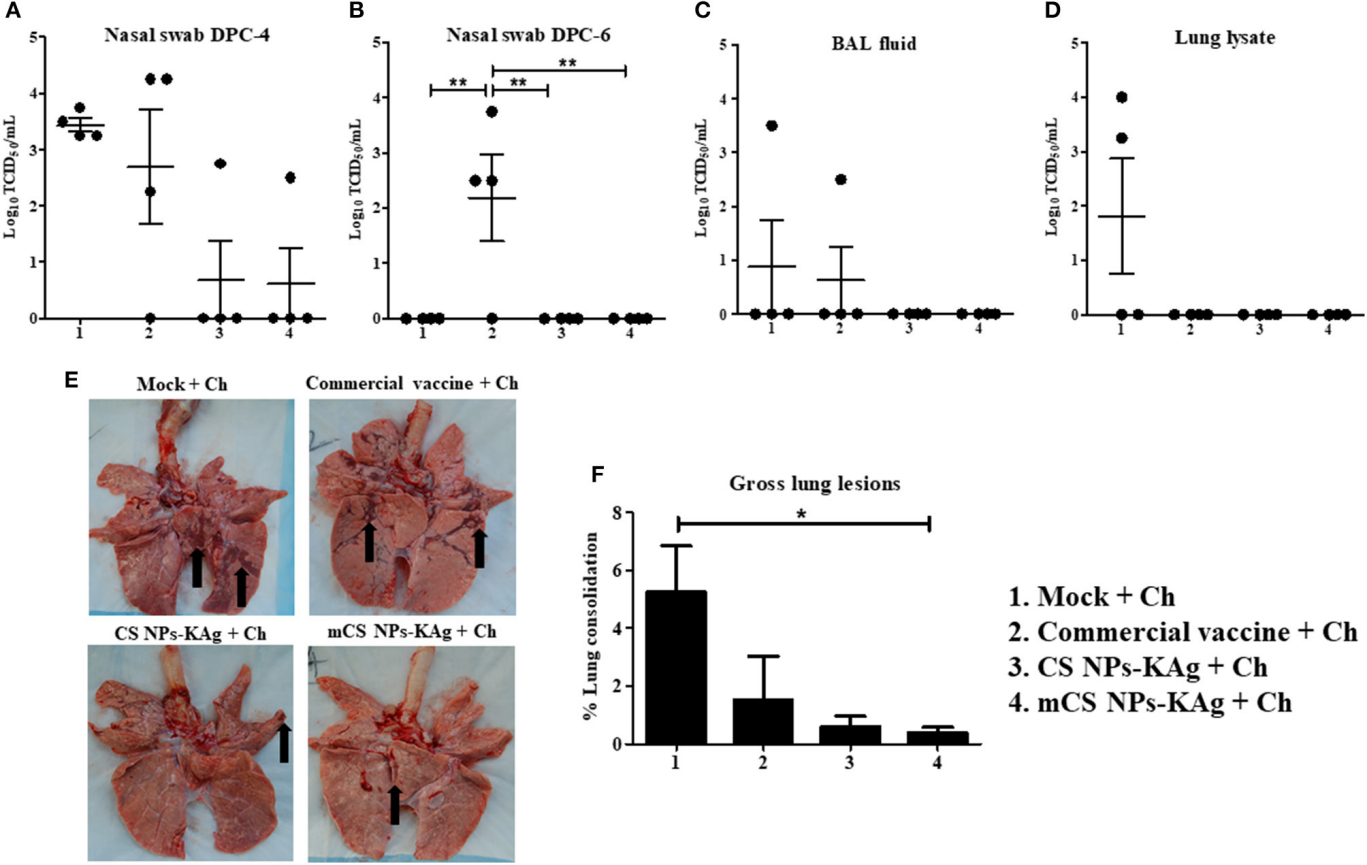
Mannose-conjugated and unconjugated CS NPs based influenza (mCS NPs-KAg and CS NPs-KAg) vaccines reduced/cleared the heterologous challenge H1N1 virus in MDA-positive pigs. Pigs were prime-boost vaccinated with mCS NPs-KAg and CS NPs-KAg vaccines (containing H1N2-OH10 virus) intranasally or commercial vaccine (containing H1N1, H1N2 and H3N2 viruses) intramuscularly and at day 35 post vaccination challenged with a heterologous H1N1-OH7 virus. At day post challenge (DPC)-4 and−6 the live H1N1-OH7 virus titers were analyzed in **(A,B)** Nasal swab; **(C)** BAL fluid (DPC-6); and **(D)** Lung lysate (DPC-6). **(E)** Representative ventral lung picture of experimental pigs showing the dark brown sites of consolidation indicated by arrows. **(F)** Macroscopic lung lesions score. Data represent the mean value of three to four pigs ± SEM. Statistical analysis was carried out using one-way ANOVA followed by Tukey's *post hoc* comparison test. Asterisk refers to statistical difference between the two indicated groups (*p < 0.05, and **p < 0.01). Ch, Challenge.

To evaluate whether the mCS NPs-KAg and CS NPs-KAg vaccines-induced cross-reactive antibodies in nasal passage and lungs were translated into the cross-protection, we examined H1N1-OH7 challenge virus titers in the airways. The virus load in nasal swab at DPC-4 was completely cleared in 3 of 4 pigs vaccinated with mCS NPs-KAg and CS NPs-KAg vaccines (Figure 6A). While both mock-challenge and commercial vaccine received MDA-positive pigs had a higher virus load at DPC-4 in the nasal passage (Figure 6A). Six days after challenge (DPC-6), infectious virus was absent in mock-challenge, mCS NPs-KAg and CS NPs-KAg vaccinates and the data was significant (*p* < 0.01) compared to commercial vaccinates (Figure 6B). The challenge virus was undetectable in BAL fluid of mCS NPs-KAg and CS NPs-KAg vaccine inoculated animals, whereas one pig in both mock-challenge and commercial vaccine group had higher virus titer (Figure 6C). In the mCS NPs-KAg, CS NPs-KAg, and commercial vaccines received pigs at DPC-6 the replicating virus in lung lysate was undetectable, while higher virus titer was noticed in two of the mock-challenge animals (Figure 6D). Even though, we did not observe any visible clinical signs in any of the virus challenged pig groups, consistent with the data of virus load in the airways, both the mCS NPs-KAg and CS NPs-KAg vaccinates had reduced macroscopic gross lung lesions (Figures 6E,F), with mCS NPs-KAg group data significantly (*p* < 0.05) lower compared to mock-challenge animals (Figure 6F).

## Discussion

Inactivated influenza virus vaccine administration by the parenteral route is frequently ineffective due to the presence of MDA in weaned animals. As a result, effective protection is absent by vaccine-induced immune responses (13, 15, 16). Vaccination of pigs in the presence of MDA negatively impacts the vaccine efficacy, leads to lengthening of the clinical signs and development of SwIAV infection induced pneumonia (14, 39). Mostly, MDA is the IgG class antibody primarily transferred from mothers to offspring through colostrum. Predominantly MDA influence the vaccine-induced IgG but not local sIgA antibody production in young piglets (16). Further, mucosal vaccines elicit sIgA antibody responses that are independent by MDA-IgG levels in blood (16, 40).

The natural mucoadhesive properties of CS NPs have been widely used for vaccine delivery to mucosal surfaces (21, 24). CS NPs-based vaccines administered IN enhance immunogenicity of entrapped antigens and elicit robust cross-reactive sIgA antibody response (26, 41). In mice, mannose ligand modified CS NPs administered mucosally specifically targets and delivers loaded antigen to the DCs (42). In this study, our objectives were to evaluate the mCS NPs-KAg vaccine-induced immune responses and protection in MDA-positive pigs delivered IN, and to compare the efficiency with CS NPs-KAg vaccine and parenterally administered multivalent commercial inactivated SwIAV vaccine.

Both mCS NPs-KAg and CS NPs-KAg vaccines delivered IN to MDA-positive pigs enhanced the cross-reactive sIgA in nasal passage, and sIgA and IgG antibodies in lung lysate and BAL fluid compared to the commercial vaccine. Particularly, mCS NPs-KAg vaccine compared to CS NPs-KAg vaccine induced increased trend (non-significant) in cross-reactive sIgA after prime-boost vaccination in nasal passage, and after virus challenge both the sIgA and IgG antibodies responses were significantly increased in BAL fluid. The IM-administered commercial vaccine increased cross-reactive IgG antibody responses and the sIgA antibody level was lower than mCS NPs-KAg vaccine. Consistent with our previous study in MDA-negative pigs (35) the commercial SwIAV vaccine did not induce significantly higher mucosal antibody responses in MDA-positive animals. In another study, adjuvanted influenza whole-inactivated virus administered pigs by IM route in both MDA-positive and -negative pigs there was absence of sIgA antibody response (15).

A study in mice has revealed that protein antigen loaded mannosylated chitosan microspheres binds with MR on macrophages, and in mice IN vaccinated, it induces high levels of antigen specific sIgA antibody response (43). Mannose modified CS NPs based vaccine promotes maturation and antigen presentation ability of DCs *in vitro*, and *in vivo* facilitates uptake of antigens by endogenous DCs within the draining lymph nodes in mice (29). Mannosylated nanoparticles internalized by macrophages induce its maturation through activation of MHC class I and II molecules *in vitro* (44).

In our recent studies (24, 25), the CS NPs preparation method was optimized and achieved a monodispersed particle of uniform size and high positive surface charge. A similar method was adapted to prepare both the mCS NPs-KAg and CS NPs-KAg vaccines which induced both cross-reactive sIgA and IgG antibodies in the lungs. In an earlier study (26), polydispersed and particles carrying relatively less positive surface charge on CS NPs-KAg vaccine induced a lesser cross-reactive IgG antibody response in IN vaccinated pigs, suggesting that size and charge of CS NPs-KAg vaccine are important to elicit a broader immune response. Further, in contrast to our present study, the optimized CS NPs-KAg vaccine co-delivered with a Th1 response promoting adjuvant poly(I:C) did not induce higher cross-reactive mucosal immune responses in pigs (24), suggesting the proper selection of secondary adjuvant is critical to achieve robust mucosal immune responses. Both in mice and chickens, CS NPs based influenza vaccine administered IN increased systemic IgG and mucosal sIgA antibodies (45, 46). Our study in MDA-positive young swine confirmed the potent immunogenicity of mCS NPs-KAg and CS NPs-KAg vaccines when delivered IN resulting in increased breadth of mucosal and systemic immune responses.

In this study, both the mCS NPs-KAg and CS NPs-KAg vaccines in MDA-positive pigs increased the vaccine (H1N2-OH10) virus-specific serum HI titers, results similar to MDA-negative swine given CS NPs-KAg vaccine (26). Likewise, CS NPs-KAg and poly(I:C) co-administration IN in pigs increased the H1N2-OH10 virus specific serum HI titer (24).

In lung draining TBLN, both mCS NPs-KAg and CS NPs-KAg vaccines increased the recall cell proliferation, and especially the former augmented the cytokine (IL-4, IL-10, and IFNγ) gene expression. Consistent with the present study, in another vaccine trial (35) the commercial SwIAV vaccine did not augment Th1 and Th2 cytokines gene expression. Studies have shown that influenza virus-specific effector T cells help in the clearance of virus by triggering the expression of cytokines IFNγ, TNFα, IL-4, and IL-10 (47–49). As well, upon reinfection memory cells produced the effector T cells to facilitate control of the infection (50).

Chitosan enhances T cell responses by promoting the maturation of DCs by signaling nascent DCs in a type I IFN receptor-dependent manner (51). Co-immunization of CS NPs-KAg and poly (I:C) vaccine enhanced the Th1 and Th2 cytokines gene expression in pigs (24). Mannan conjugated antigen hasten the MHC class I presentation to CD8^+^ T cells, leads to Th1 immune response (52). The Th2 cytokines IL-4 and L-10 are the driving factor to upregulate MR expression in macrophages (53), and higher MR expression is associated with the induction of Th2 mediated immune response (54).

The mCS NPs-KAg and CS NPs-KAg vaccines enhanced cross-reactive sIgA and IgG antibody levels in both the nasal passage and lungs (lung lysate and BAL fluid), which, together with increased cellular responses, resulted in reduced/cleared challenge heterologous (H1N1-OH7) SwIAV from both the upper and lower respiratory tract. Furthermore, mCS NPs-KAg and CS NPs-KAg vaccines reduced the influenza virus titers correlated with decreased macroscopic lung lesions. Earlier studies have established that circulating sIgA and IgG antibodies induced by an IN vaccination has been correlated with protection against influenza virus infection in mice, chicken, pigs, and humans (26, 45, 46, 55). Antigen specific sIgA antibody more efficiently prevents the influenza virus infection of mucosal surfaces than does circulating systemic IgG antibody (56, 57). The innate and cell-mediated immunity also plays a key role in the clearance of influenza viral infection from infected tissues (58, 59).

Consistent with an earlier study (39), commercial inactivated influenza virus vaccine in the present study did not provide a protection of upper respiratory tract infection in pigs. An IM immunization of commercial inactivated influenza virus vaccine in MDA-positive pigs boosts HI titer, but not sIgA and indices of the cellular immune responses and thus failed to provide protection against heterologous virus infections (39). A study has shown that adjuvanted whole inactivated influenza virus vaccine administered IM in MDA-positive pigs dramatically increased a phenomenon known as vaccine-associated enhanced respiratory disease following heterologous virus challenge (14).

## Conclusions

The mannose conjugated CS NPs delivered monovalent inactivated SwIAV vaccine administered IN in MDA-positive pigs augmented the homologous, heterologous, and heterosubtypic virus specific mucosal sIgA and IgG and systemic IgG antibodies in airways. Both CS NPs-KAg vaccines, especially the mCS NPs-KAg vaccine, increased specific recall cell proliferation and cytokine gene expression in the tracheobronchial lymph nodes resulting in reduced/cleared heterologous challenge virus infection. Overall, our study results suggested that mCS NPs-KAg vaccine IN delivery could be a useful and effective alternative to commercial influenza vaccine for inducing cross-protective immunity against SwIAVs in MDA-positive grower finisher pigs.

## Data Availability Statement

The raw data supporting the conclusions of this article will be made available by the authors, without undue reservation.

## Ethics Statement

The animal study was reviewed and approved by The Institutional Animal Care and Use Committee at The Ohio State University.

## Author Contributions

SR and GR conceived, developed the research, and wrote the manuscript. SR formulated and characterized the vaccines. SR and NF did the experiments and analyzed the data. SR, NF, VP, JS, YH, and AR helped in vaccination and challenge trial in pigs, including sample collection and processing. JH helped in vaccination of sows and monitoring and procuring maternal antibody positive piglets. SK edited the manuscript. SD grew the vaccine and challenge viruses. All authors read and agreed the manuscript for publication.

## Conflict of Interest

The authors declare that the research was conducted in the absence of any commercial or financial relationships that could be construed as a potential conflict of interest.

## Supplementary Material

Supplementary Figure 1 Experimental pig groups had the influenza virus specific maternal IgG antibody in the serum. Pregnant sows were vaccinated with commercial influenza vaccine at 2 and 5 weeks before farrowing. Serum collected from weaned piglets at 3 weeks of age was serial 10-fold diluted and analyzed for the H1N2-OH10 virus specific IgG antibody level. Data represent the mean value of three to four pigs ± SEM at all indicated dilutions. Statistical analysis was carried out using one-way ANOVA followed by Tukey's *post hoc* comparison test.

Supplementary Table 1 Sequence of the primers used in qRT-PCR analyses.
