# Supplementary figures and images for: Immunity and Protective Efficacy of Mannose Conjugated Chitosan-Based Influenza Nanovaccine in Maternal Antibody Positive Pigs
Source: Front Immunol. 2021 Mar 4;12:584299. doi: 10.3389/fimmu.2021.584299 (PMC7969509; doi:10.3389/fimmu.2021.584299)

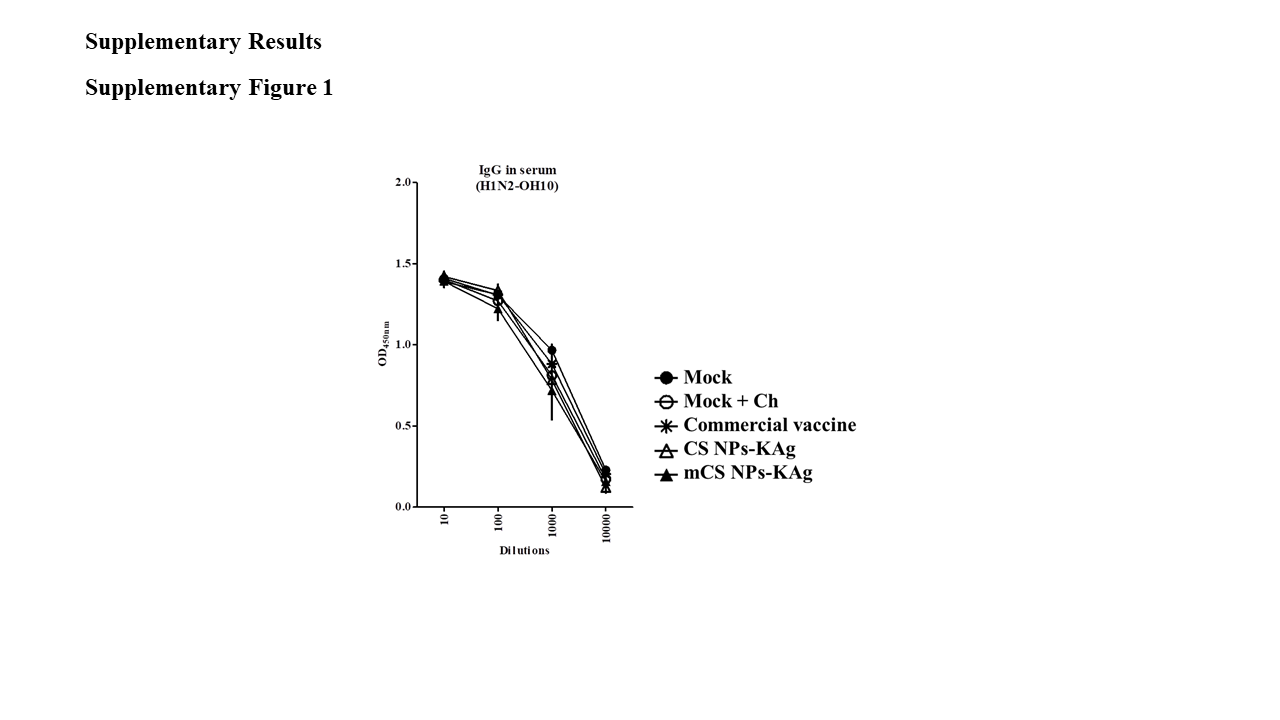

Supplement: Supplementary Figure 1 — Experimental pig groups had the influenza virus specific maternal IgG antibody in the serum. Pregnant sows were vaccinated with commercial influenza vaccine at 2 and 5 weeks before farrowing. Serum collected from weaned piglets at 3 weeks of age was serial 10-fold diluted and analyzed for the H1N2-OH10 virus specific IgG antibody level. Data represent the mean value of three to four pigs ± SEM at all indicated dilutions. Statistical analysis was carried out using one-way ANOVA followed by Tukey's post hoc comparison test. [file Image_1.TIFF]
