# Supplementary material for: Immunity and Protective Efficacy of Mannose Conjugated Chitosan-Based Influenza Nanovaccine in Maternal Antibody Positive Pigs
Source: Front Immunol. 2021 Mar 4;12:584299. doi: 10.3389/fimmu.2021.584299 (PMC7969509; doi:10.3389/fimmu.2021.584299)
Supplement: Supplementary Table 1 — Sequence of the primers used in qRT-PCR analyses. [file Table_1.DOCX]

**Supplementary Table S1**

| **S. No** | **Oligo name** | **Sequence (5’ 3’)** |
| --- | --- | --- |
| 1 | *β-actin* | CAGCCTCCTGAAACTGGAATAT (F)  TCAGCAACAAGGTCTACAATCC (R) |
| 2 | *IL-4* | ATCCCAACCCTGGTCTGC (F)  TCCTGTCAAGTCCGCTCA (R) |
| 3 | *IL-10* | GCATCCACTTCCAGGCCA (F)  CTTCCTCATCTTCATCGTCA (R) |
| 4 | *IFN-γ* | CAGCTTTGCGTGACTTTGTG (F)  GATGAGTTCACTGATGGCTTT (R) |
